# Epidemiology, Diagnosis, Treatment, and Prognosis of Infective Endocarditis

**DOI:** 10.3390/jcm12175705

**Published:** 2023-09-01

**Authors:** Manuel Martínez-Sellés, Patricia Muñoz

**Affiliations:** 1 Instituto de Investigación Sanitaria Gregorio Marañón, 28009 Madrid, Spain; pmunoz@hggm.es; 2 School of Medicine, Universidad Complutense de Madrid, 28040 Madrid, Spain; 3 Cardiology Department, Hospital General Universitario Gregorio Marañón, CIBERCV, Universidad Europea, 28001 Madrid, Spain; 4 Department of Clinical Microbiology and Infectious Diseases, Hospital General Universitario Gregorio Marañón, 28007 Madrid, Spain; 5 CIBER Enfermedades Respiratorias-CIBERES (CB06/06/0058), 28029 Madrid, Spain

Infective endocarditis (IE) has experienced enormous changes in recent decades. This Special Issue in the *Journal of Clinical Medicine (JCM)* is dedicated to the epidemiology, microbiology, diagnostics, and treatment of IE.

IE is a disease with a poor prognosis, whose diagnostic criteria have been recently updated [1] to include new microbiology [2] and imaging diagnostics [3] and additional predisposing conditions such as transcatheter valve implants [4] and cardiac implantable electronic devices [5]. We are witnessing a radical modification of the paradigm of IE. The present and past differ in the type of affected population, risk factors that predispose to it, place of acquisition, etiology, medical management, and surgical treatment. A single doctor frequently managed classical IE. The physician suspected the disease when young patients with previous cardiac rheumatic fever developed a fever and new murmurs. The most common etiology was *Streptococcus viridans*, and the condition, before penicillin was available, was almost uniformly deadly. Today, most patients have advanced age and previous heart disease, frequently with valvular prostheses and electronic cardiac devices, along with multiple comorbidities. The incidence of IE is about 5 per 100,000 people per year. Echocardiography and positive blood cultures (frequently for *Staphylococcus* spp.) are usually the keys to the diagnosis, but a growing list of imaging and microbiological techniques is now available. Surgery is part of the solution for about half of IE patients, and, although mortality continues to be high, most patients survive.

In any case, IE remains a difficult condition to diagnose and treat. Multidisciplinary endocarditis teams are now considered essential in its management [6]. All patients with suspected or confirmed endocarditis should be managed in consultation with a multidisciplinary team that includes, at least, infection specialists, imaging experts, cardiologists, and cardiac surgeons. Other relevant specialties, including neurology, nephrology, pediatrics, geriatrics, anesthesiology, and critical care, should be readily available to the endocarditis team. The team’s role is to perform a rapid and accurate diagnosis, prescribe appropriate antibiotics, refer patients to surgery if required, arrange and interpret imaging data, and follow up with patients after discharge.

The epidemiology of IE has also changed dramatically, mainly due to population aging. Most patients now have multiple comorbidities, and there has been a clear increase in nosocomial and healthcare-related IE in recent decades. Perez-Rivera et al. [7] describe a national cohort of octogenarians with IE and find that half of them have high comorbidity. Moreover, high comorbidity was a strong independent predictor of in-hospital and 1-year mortality in this elderly population. In addition, the data presented strongly suggest that the underperformance of cardiac surgery in this group of elderly patients may play a role in their poor prognosis.

According to Biezma et al. [8], the prevalence of diabetes mellitus among patients with IE is increasing and has already surpassed 30%. This is consistent with the fact that diabetes is quickly becoming the most serious epidemic of the twenty-first century, and its prevalence is expected to rise in the coming years. New estimates indicate that more than 1.3 billion people may have diabetes by 2050 [9]. In the study presented by Biezma et al. [8], diabetes was not only prevalent but was also independently associated with a poor prognosis, particularly in the case of diabetes with organ damage.

Musci et al. [10] review healthcare-associated IE from a surgical perspective. Though definitions vary, in general, healthcare-associated IE includes IE acquired 48 h after hospital admission or associated with a significant invasive procedure performed 6 months prior to clinical diagnosis. Healthcare-associated IE already accounts for half of all cases and is expected to grow in the near future. Advanced age, cardiac implants, and comorbidity are important predisposing factors, and intravascular catheters or frequent vascular access are significant sources of infection. As expected, staphylococci and enterococci are the leading causative microorganisms of healthcare-associated IE. Despite being frequently indicated, surgery is rejected by approximately half of the patients due to the prohibitive risk. After surgery, in-hospital mortality is high (29–50%), but it is higher in rejected patients (52–83%). This entity should be recognized at the time of admission rather than being treated as a community-acquired IE.

Cecchi et al. [11] present the data from the Italian Registry of IE in people who inject drugs, a predisposing condition that used to be very common. So far, we have primarily received recent information from the United States regarding the American opioid pandemic, so European data are greatly appreciated. From a total of 677 enrolled patients, 9% were intravenous drug users, with an overall mortality of 10% and 20% in patients with recurrent IE. IE in intravenous drug users mostly affected native valves (90%); *Staphylococcus aureus* was the main microorganism (70%); and about half of the patients underwent heart surgery. Overall, 34% of the patients tested positive for HIV, and 33% of them had a CD4 level <200/mm^3^.

The best treatment for each type of IE is frequently unknown. Given the increasing age and comorbidity of patients with IE, it is essential to be very aware of the significance of protecting patients from treatment toxicity such as aminoglycoside nephrotoxicity. In this regard, it is of the utmost importance to disseminate the high efficacy of the combination of two beta-lactams, ampicillin and ceftriaxone, to treat *E. faecalis* IE. Herrera-Hidalgo et al. [12] describe the efficacy and safety of three ampicillin-plus-ceftriaxone regimens for *Enterococcus faecalis* IE in 59 outpatients who received parenteral antibiotic treatment. Two grams of ampicillin every 4 h plus 2 g of ceftriaxone every 12 h (the preferred inpatient regimen) and ceftriaxone co-diluted and jointly administered with ampicillin every 4 h showed similar results, but once-daily high-dose administration of the ceftriaxone produced an unexpected rate of relapses. Six relapses were observed in the entire cohort, with five patients (29%) receiving once-daily high-dose administration of the ceftriaxone regimen and one patient (10.0%) receiving ampicillin plus ceftriaxone co-diluted and jointly administered in a bolus every 4 h. In conclusion, once-daily high-dose administration of ceftriaxone exhibited an unexpected rate of failures; however, ampicillin plus ceftriaxone co-diluted and jointly administered in a bolus every 4 h might be an effective alternative for outpatient parenteral antibiotic treatment.

The optimal timing for cardiac surgery in IE is uncertain, particularly in patients with neurological complications. Approximately a quarter of patients will present either focal signs due to embolisms, focal signs due to abscesses or mycotic aneurysms, meningeal syndrome due to hematogenous seeding of the central nervous system, or encephalopathy secondary to sepsis. Siquier-Padilla et al. [13] review this topic and conclude that cardiac surgery can be performed without delay in cases of ischemic, infectious, or asymptomatic neurological complications. In the presence of intracranial hemorrhage, a delay of four weeks is recommended for most cases.

Despite recent advances, the IE outcome remains poor. More research is needed, particularly in uncommon subgroups such as children [14,15], pulmonary IE [16], multivalvular IE [17], patients with solid organ transplantation [18,19], bicuspid aortic valves [20], and those treated with oral therapy [21]. In addition, better prognostic markers [22,23], more specific imaging techniques [24], and improved surgical scores [25] that aid in clinical decision-making are required. IE continues to be a disease with high morbidity and mortality rates. The combination of clinical, microbiological, and cardiac imaging evaluations is essential for the early diagnosis and risk stratification of IE. An early diagnosis is key to improving the outcomes of medical and surgical therapies. Recent laboratory and imaging modalities advances provide complementary IE diagnostic and prognostic information. IE is a severe, multisystem disease that often requires cardiac surgery. IE is becoming more common in patients with intracardiac prosthetic valves and devices. Timely and appropriate blood culture sampling and echocardiography are essential for diagnosis. This Special Issue aims to present IE in specific populations and discuss the best way to assess and treat IE in various subgroups.
